# Investigation of Friction and Wear Behavior of Cast Aluminum Alloy Piston Skirt with Graphite Coating Using a Designed Piston Skirt Test Apparatus

**DOI:** 10.3390/ma15114010

**Published:** 2022-06-05

**Authors:** Dezhi Teng, Jingsi Wang, Chengdi Li, Xiaoxia Sa

**Affiliations:** 1 Marine Engineering College, Dalian Maritime University, Dalian 116026, China; z15966667693@163.com; 2 Xinyu Key Laboratory of Materials Technology and Application for Intelligent Manufacturing, Xinyu University, Xinyu 338004, China; lichengdi17@163.com; 3 Department of Materials Science and Engineering, Dalian Maritime University, Dalian 116026, China; sa_empty@163.com

**Keywords:** friction and wear tester, piston skirt, coating, wear, plateau

## Abstract

A piston skirt friction and wear apparatus that simulates the contact and the relative motion of piston and cylinder liner in a real engine has been designed and constructed. With this apparatus, the friction and wear behavior of a cast aluminum alloy piston with a graphite coating under different loads was studied, and the effectiveness of the apparatus was confirmed. The total wear of the piston skirt was higher under a higher load, and the upper part of the skirt surface (around the height of the piston pin) was worn more severely. The wear mechanisms were studied and, based on the test results and surface analyses, three main wear modes were believed to occur in the wear process of the piston skirt: abrasive, adhesive, and fatigue wear. The effects of skirt profile design, coating, and surface texturing on the friction and wear behavior of the piston skirt can be investigated well using the proposed apparatus, which can truly reflect actual working conditions and is useful to improve the tribological performances of piston skirts.

## 1. Introduction

The development in high-power density of internal combustion engines has led to increasing attention being paid to the wear-resistance of key friction pairs, which directly affect the reliability, efficiency, and energy consumption of diesel engines [1,2]. Three major subsystems of the internal combustion engine, contributing to mechanical friction, are the piston-cylinder system, the crankshaft-bearing system, and the valve-train system [3]. Especially relevant is the fact that about 50% of the total wear loss results from the piston-cylinder system. In the piston-cylinder assembly, the piston skirt, as a critical engineering feature, is mainly used for absorbing side thrust loading resulting from combustion forces and inertia forces of rotating parts, stabilizing piston motion within the cylinder bore and improving the ring seal, by preventing the piston from rocking too much. Generally, increased clearance between the piston and the cylinder liner resulting from wear of the piston skirt is harmful to oil consumption and engine noise. Therefore, the friction and wear behavior of the piston skirt has a large effect on the performance of a piston and has become a focus point in the piston-cylinder system [4].

**Figure 1:**
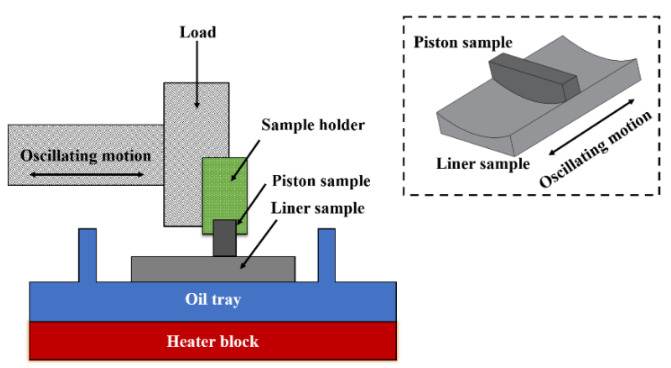
Schematic of the setup and the contact conditions between material pairs in a wear test of piston and cylinder liner (Adapted from Refs. [12,13]).

A significant number of theoretical analyses considering the surface profile, elastic deformation of pistons and lubrication conditions have been conducted to study friction and wear properties between the piston skirt and cylinder liner [5,6,7,8]. Furthermore, the inertia effect and varied dynamic conditions have also been studied in some models to predict piston-liner dynamic behaviors and friction characteristics [9,10,11].On the other hand, limited experimental work has been conducted to explore the friction and wear behavior of piston skirt-cylinder liner assembly, compared to theoretical studies. Wang et al. [12,13] conducted wear tests of coated piston samples (6 × 22 mm^2^) against varied cylinder samples (58 × 20 mm^2^) with a high frequency friction machine. The samples of pistons and cylinders were cut from commercial products. The schematic of the setup and the contact condition between the piston and cylinder liner samples are shown in Figure 1. Demas et al. [14] investigated tribological performances of different coated piston skirts mating with commercial grey cast iron cylinder segments, and the tests used commercial synthetic motor oil. A faint tribological film appeared on the cylinder surface because the anti-wear additives in the lubrication oil were thermally activated during sliding. Shaw et al. [4] studied tribological performances of piston skirt coatings using two test configurations: one was conformal contact to study friction behavior under normal operating conditions, and the other one was point contact to investigate wear behavior under extreme pressure conditions. In the studies of Wang et al. [15] and Ye et al. [16], the scuffing resistance of coated piston skirts was experimentally investigated. Results showed that surface roughness, mechanical and chemical properties of the tribo-pairs and piston coatings, and the tribo-chemical reactions at the contact interface were the main factors influencing scuffing. All the tribo-pairs used in the above research were only small parts extracted from the piston skirt and cylinder liner components, and the impact of the skirt profile on the friction and wear behavior was ignored. Nevertheless, the skirt profile is the axial structure of the piston aimed at generating an appropriate contact area and reasonable pressure between skirt and cylinder liner when the piston is in motion [17]. The piston skirt profile, which could prevent the piston skirt scraping and maintain good lubrication between the tribo-pairs, plays a vital role in the secondary motion of the piston. Thus, friction and wear tests with sample pieces cannot truly reflect actual working conditions, which is not helpful for a total understanding of the tribological behavior of the piston skirt. Up to now, there has been little published information on friction and wear tests of engine friction pairs using whole engine parts, except for bench tests. Bench tests were performed for evaluation of the wear and scuffing of piston skirts, considering the effects of piston skirt coating and pin offset [18,19,20]. However, the experiments were costly, time-consuming, and varied from case to case, as they were conducted under real ignition conditions, which limited the application of bench tests in evaluating tribological characteristics of piston skirts.

Therefore, although the friction and wear behaviors of the piston skirt have been widely studied, based on existing literature, there is still a lack of effective friction and wear testing machines and methods, and more case-by-case studies are needed, due to the complexity of tribological behavior and diversity of operational conditions. In this work, a novel piston skirt friction and wear apparatus, that simulates the contact and the relative motion of the piston and cylinder liner in a real engine, was developed to study the tribological behavior of a cast aluminum alloy piston with a graphite coating. The whole piston and cylinder liner parts were used in the experiments. The friction and wear behaviors of the piston skirt under variable loads were studied. Reported in this paper are the development of the friction and wear apparatus, methods and results of the friction and wear experiments, and discussions on the experimental results.

## 2. Experimental Apparatus

**Figure 2:**
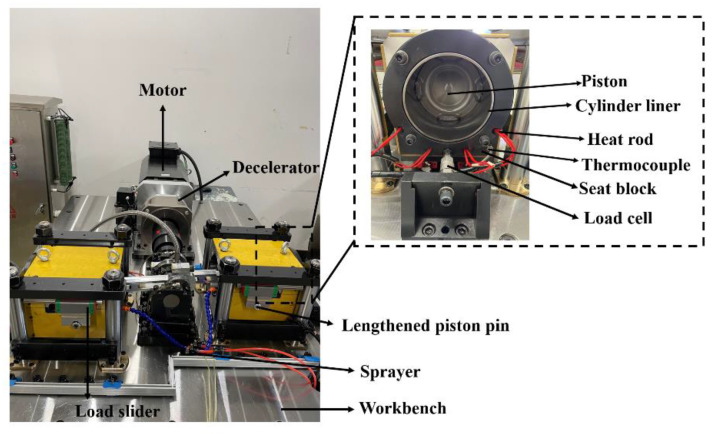
Overall appearance of the apparatus.

Design of the apparatus was based on the following considerations: (a) the contact of the tribo-pairs should simulate the worst contact under the combined effects of thermal and mechanical loads during the expansion stroke; (b) the relative motion between the tribo-pairs should be the same as that in a real engine; and (c) the lubrication condition should be similar to that of a real working status. In a diesel engine, the piston slides in the cylinder and transfers force from expanding gas to the crankshaft via the piston pin and the connecting rod. In the apparatus, a speed controllable motor was applied to drive a crankshaft electrically, and, then, to actuate the piston reciprocating in the cylinder through a piston pin and a connecting rod. The driving process is opposite. The apparatus allows for precise control of the load, temperature, sliding speed, and flow rate of the lubricating oil. Figure 2 shows the overall appearance of the apparatus. The main elements of the apparatus are detailed as follows.

### 2.1. Motion System

The friction and wear test apparatus were built symmetrically to allow the conducting of two sets of tests simultaneously, and the sliding direction was horizontal. The configuration was helpful to weaken inertial effects and to make the apparatus stable. The cylinder liner was horizontally fixed at a seat block. The piston was driven by the rotation of the crankshaft and reciprocated in the cylinder liner, and the crankshaft was driven by a spindle motor. A decelerator was connected to the motor with a reduction ratio of 5:1. The crankshaft speed was up to 800 rpm and the stroke length was 96 mm.

### 2.2. Loading and Heating System

Loading was applied through the piston pin to simulate real loading conditions. The piston pin was lengthened. Symmetrical openings were cut in advance in the sidewalls of the cylinder liner to allow the passage of the lengthened piston pin, which was finally connected to a load slider. During the experiments, the loading force was transmitted to the load slider by controlling a jack assembly, and a side thrust loading was achieved. The maximum side thrust loading applied to the piston could reach up to 100 kN.

The heating system relied on heating rods with a power of up to 600 W, which were evenly arranged in the seat block. After the heating system was started, the cylinder liner could reach the predetermined target temperature, ranging from 100 to 300 °C, in a short time.

### 2.3. Lubrication System

Two lubrication systems were designed. One was for lubricating the test tribo-pairs, which consisted of an oil bath, a regulator to control the amount of the injected oil, and a sprayer to spray oil atomized by compressed air. The design was to simulate the lubrication in a real engine where the lubricating oil is splashed on to the friction interface. The other one was for lubricating the crankshaft assembly. The lubricating oil was pumped into the through-hole in the main journal and passed through the internal channels of the crankshaft to lubricate the crankshaft-bearing friction pairs.

### 2.4. Measurement System

**Figure 3:**
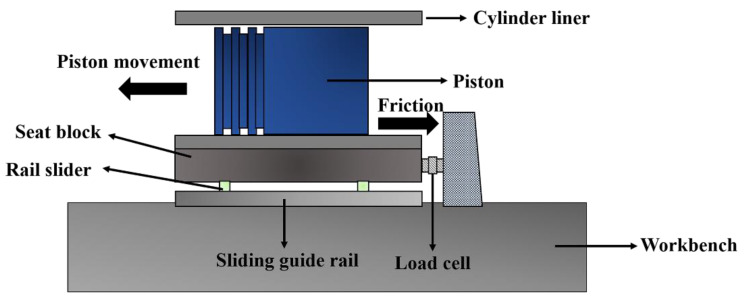
Schematic diagram of how to measure the friction force.

The load and the friction force were measured with two load cells. One load cell was fixed under the workbench to measure the side thrust loading. The other load cell was to measure the friction force, and Figure 3 shows a schematic diagram of how it worked. The seat block for fixing the cylinder liner was connected to a rail slider. The existence of friction between the piston and the cylinder liner would make the seat block tend to move. The movement tendency of the seat block was prevented by the load cell, which was fixed on the workbench, as shown in Figure 3. Accordingly, the friction force was captured. Thermocouples embedded at the inner surface of the seat block were used to directly measure the temperature.

## 3. Materials and Experimental Methods

**Figure 4:**
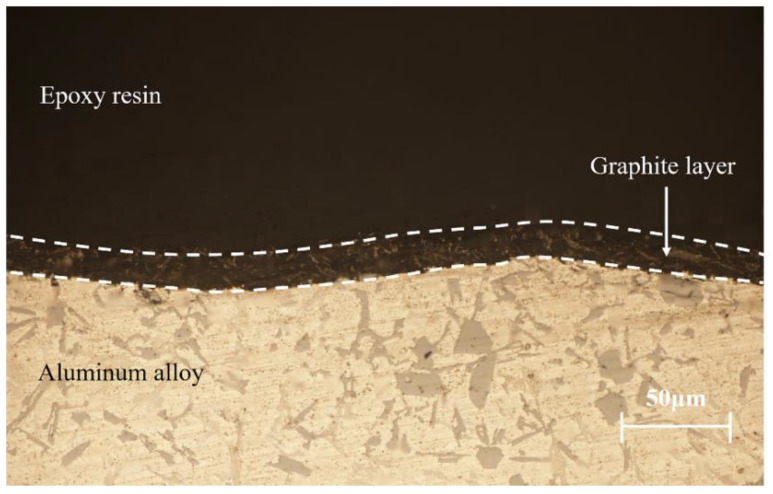
Metallographic analysis of the cross-section of the skirt.

**Figure 5:**
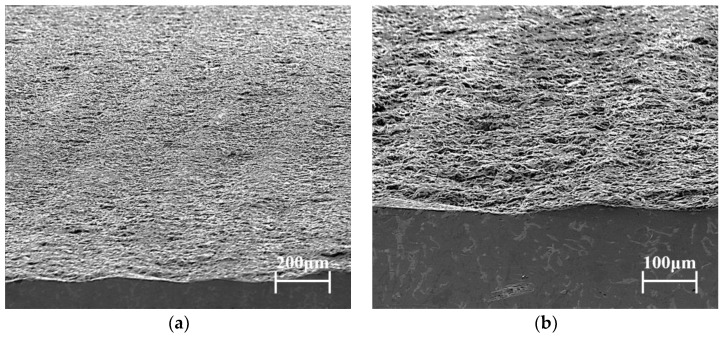
Cross-section morphologies of the skirt: (**a**) 200× magnification, (**b**) 500× magnification.

**Figure 6:**
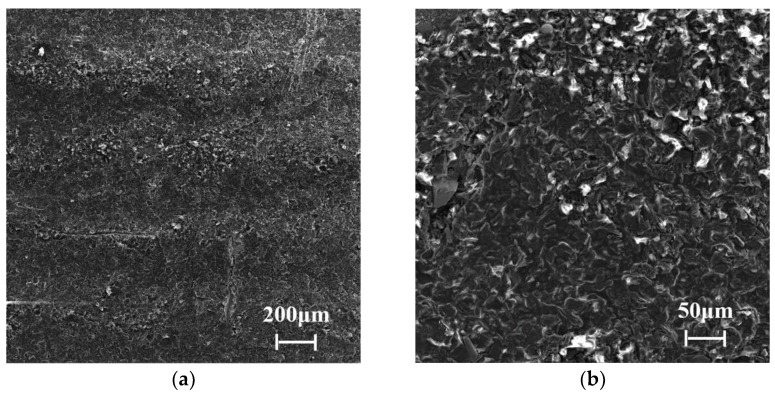
Morphologies of the skirt surface at different magnifications: (**a**) 200× magnification, (**b**) 1000× magnification.

Cast aluminum-silicon alloy pistons were used in the experiments, the chemical composition of which is listed in Table 1. The tested piston skirts were sprayed with graphite coatings using a designed sprayer after phosphating, and a metallographic analysis was performed on the cross-section, as shown in Figure 4. The thickness of the coating was about 20 μm. The hardness of the substrate material was 201.7 HV. Figure 5 and Figure 6 show the cross-section and surface morphologies of the piston skirt, using scanning electron microscopy, respectively. The surface was designed to have a concave–convex profile with a peak-to-peak spacing of about 300 μm, which is believed to be helpful for lubricating oil retention under mixed or boundary lubrication conditions [16]. This design has also been proven to have excellent fuel economy and to reduce the risk of scuffing [21]. Additionally, graphite flakes were evenly stacked on the skirt surface.

**Figure 7:**
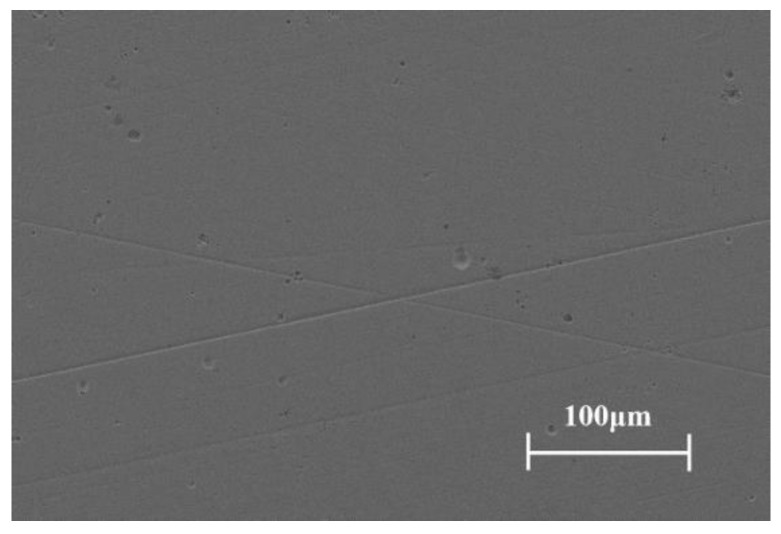
SEM micrograph of the cylinder liner surface.

The cylinder liner as the mating pair was made of 38CrMoAl steel and had a mirror-like finish. The surface was processed with nitriding treatment. The inner diameter of the cylinder liner was 150 mm. The surface hardness and average surface roughness Ra of the cylinder liner was 820.4 HV and 0.2 μm, respectively. Figure 7 shows the SEM micrograph of the liner surface. Honing marks with a cross-hatch angle of 25° can still be found on the surface.

**Figure 8:**
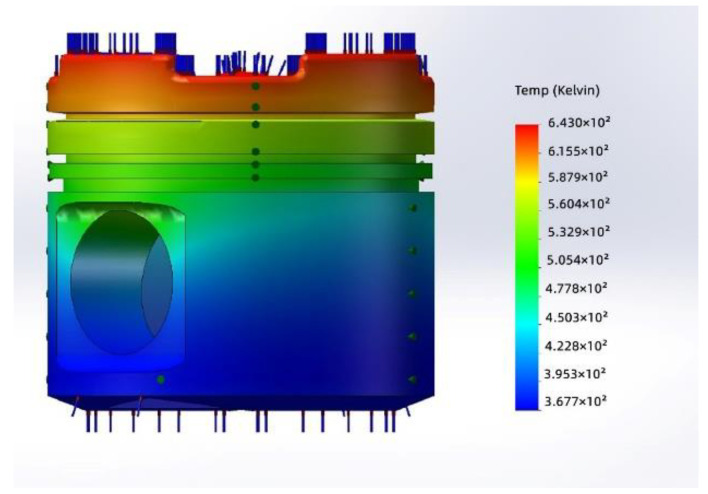
Temperature distribution of the piston skirt.

The temperature distribution of the piston skirt, based on a typical bench test, is shown in Figure 8. It was found that the temperature of the piston skirt ranged from 90 to 150 °C. Accordingly, the temperature was set to 120 °C for the friction and wear test in this study.

The side thrust force applied to the piston skirt was determined by the following equation [22]:

(1) $$P_{h}=[\frac{{\pi \cdot D}^{2}}{4}p-p_{0}-m_{j}(R\omega^{2}\cos\alpha +R\omega^{2}\cos\alpha )\frac{\mathrm{Rsin}\alpha }{\sqrt{L^{2}-R^{2}\sin^{2}\alpha }}]$$

where *P_h_* is the side thrust load applied to the piston skirt, *D* is the diameter of the piston, *p* is the gas pressure in the cylinder liner, *p*_0_ is the gas pressure in the crankcase, which is about 0.1 MPa, *m_j_* represents the weight of the piston and piston pin, *R* is the radius of the crankshaft, *ω* refers to the rotation speed of the crankshaft, α is the crank angle of the maximum combustion pressure in the cylinder liner, and *L* is the length of the connecting rod. The calculated side thrust force on the piston skirt was about 10 kN during the expansion stroke, according to equation (1). In the wear experiment, three different loads of 8, 10 and 12 kN were tested to study the friction and wear behavior of the piston skirt. As the load existed during the whole test, wear of the piston skirt would be accelerated, which was helpful to study the friction and wear behavior in a short time.

CF4 10W-40 diesel oil was used as the lubricating oil. The kinematic viscosities of this oil were 15.33 and 86.06 cSt at 100 °C and 40 °C, respectively. This lubricating oil contains anti-wear additives of zinc dialkyldithiophosphate (ZDDP). The friction and wear tests were conducted for a total of 40 h, which consisted of a running-in period with a light load and the wear period with the target load. The crankshaft speed and the flow rate of the lubricating oil were set to 200 rpm and 2 mL/min, respectively. The experimental parameters are presented in Table 2.

The outer diameters of the piston skirt at different heights from the bottom of the piston were measured every 8 h by an outer micrometer with a measurement resolution of 1 μm. The variation of the outer diameter was used to evaluate the wear of the piston skirt.

## 4. Results and Discussions

### 4.1. Wear of the Piston Skirts under Different Loads

**Figure 9:**
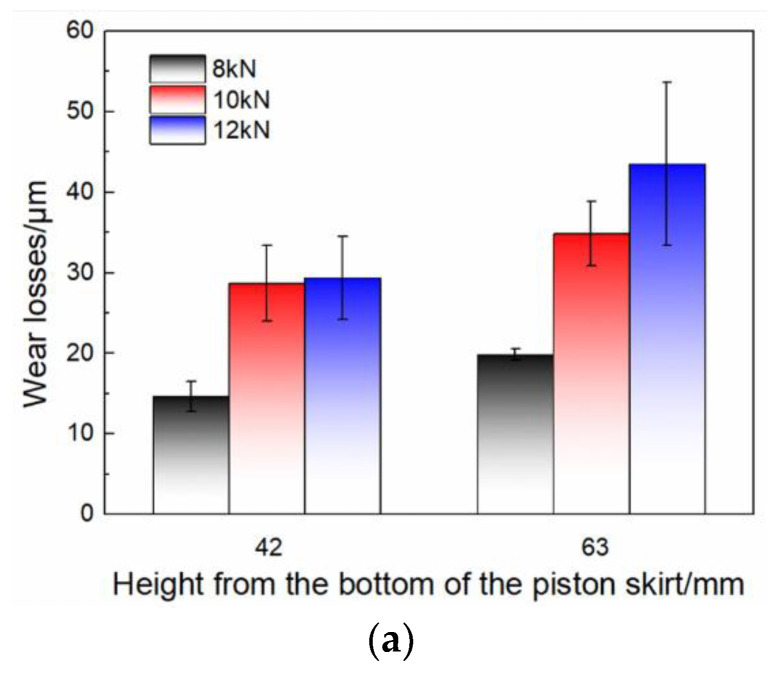

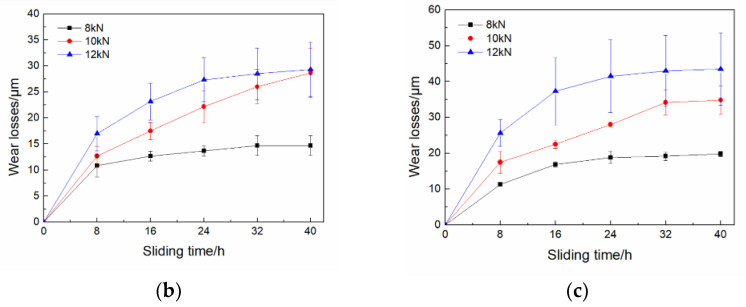
Wear of the piston skirts under different loads: (**a**) total wear of the piston skirts at varied heights, (**b**,**c**) wear of the piston skirts versus time at the height of 42 and 63 mm, respectively.

As the load existed during the whole test, wear of the piston skirt accelerated. Total wear of the piston skirt under different loads after the 40 h test is shown in Figure 9a. The results at heights of 42 and 63 mm from the bottom of the pistons are depicted. The center position of the piston pin hole was near the height of 63 mm. It was found that the wear of the piston skirt increased with increase of the load. Figure 9b,c show the wear results versus time at the two different heights, respectively. Wear increased quickly at the initial stage and then tended to be stable for both heights. It was also noted that wear was more stable after 8 h under 8 kN compared to 10 and 12 kN. On the other hand, the results showed that the skirt surface around the height of the piston pin (63 mm) was worn more severely than the lower part (42 mm) under all three different loads, and similar wear phenomena was reported in previous research [19,20]. The position perpendicular to the piston pin is a major part for bearing force, therefore the contact pressure was high in this area, and the wear was serious. It was confirmed that the current apparatus simulated the loading conditions of the real engine well and provided comparable wear results.

### 4.2. Coefficient of Friction

**Figure 10:**
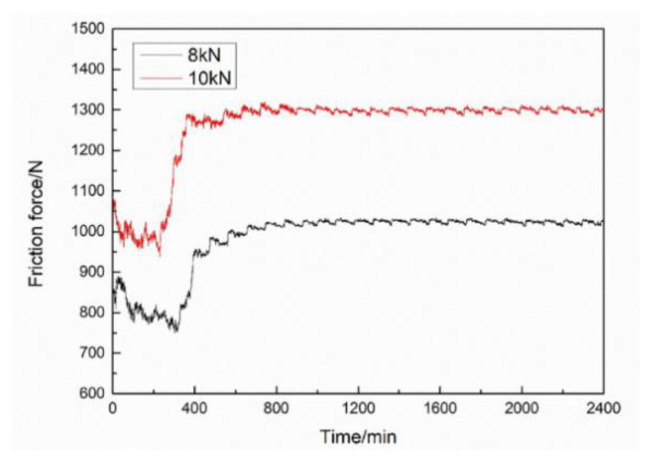
Friction forces of the tribo-pairs under varied loads.

The friction force at dead points of the tribo-pairs under 8 and 10 kN, as a function of time, is shown in Figure 10. It is obvious that the force was higher with a higher load. After the running-in stage (1/3 h), friction force increased for both 8 and 10 kN because a high target load was applied. Then, the friction force decreased quickly and remained stable for a while. After about 400 min, a sudden increase in friction force occurred in both cases, and it was a little earlier for 10 kN. After that, the friction force remained stable until the end of the friction and wear test. The low friction force during the first 400 min is considered to be due to the lubrication effect of the graphite coating on the skirt. A sudden increase of friction force means a partial removal or degradation of the solid lubricant films, which is mainly caused by adhesive shearing [23,24]. Generally, solid lubricants, including graphite and molybdenum disulfide, can efficiently improve anti-friction behavior, wear resistance and anti-scuffing capacity during the cold start of the engine. The coefficient of friction was calculated using the ratio of the friction force to the side thrust load. The highest and the lowest coefficient of friction were 0.125 and 0.094 under 8 kN, while they were 0.129 and 0.095 under 10 kN, respectively. The tribo-pairs at dead centers were under boundary lubricated conditions during the wear process, which simulated the lubrication condition of a real engine well. The above friction and wear results are consistent with existing literature and verify the effectiveness of the proposed apparatus.

### 4.3. Worn Morphology of the Piston Skirt

**Figure 11:**
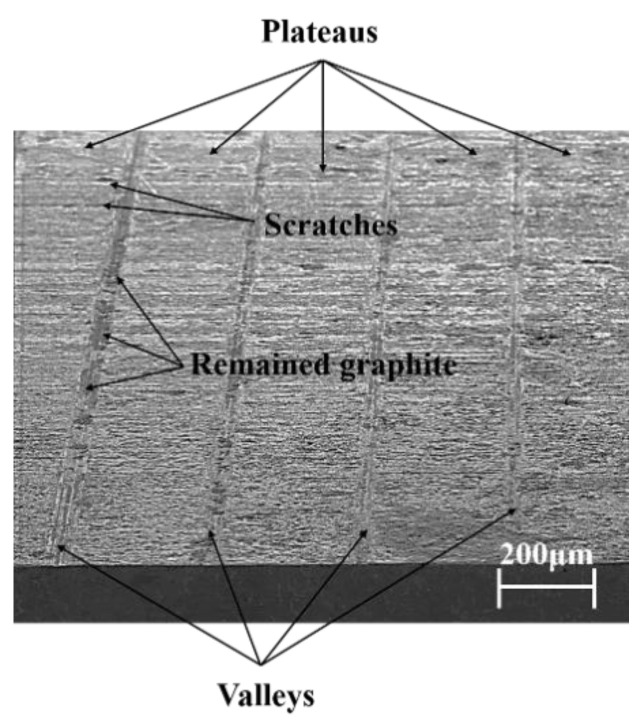
Morphology of skirt plateaus under 10 kN.

After 40 h of the friction and wear test, the worn piston skirt samples were extracted by wire-cut electrical discharge machining and observed using a scanning electron microscope. Figure 11 shows the morphology of the severely worn area of the piston skirt under 10 kN. It is obvious that platforms for bearing, which are called plateaus from the former work [25], were generated instead of the original asperity peaks (as shown in Figure 5), while valleys could still be observed. A small amount of graphite coating still remained in the valleys, and scratches along the sliding direction could be confirmed.

**Figure 12:**
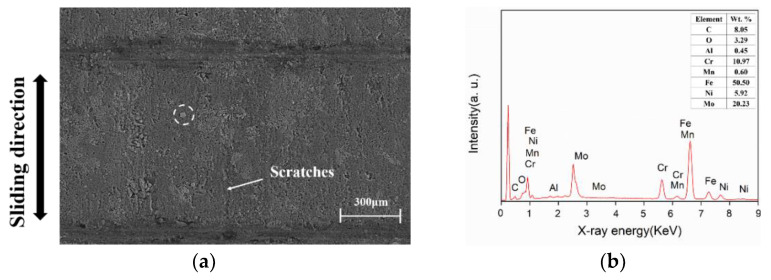

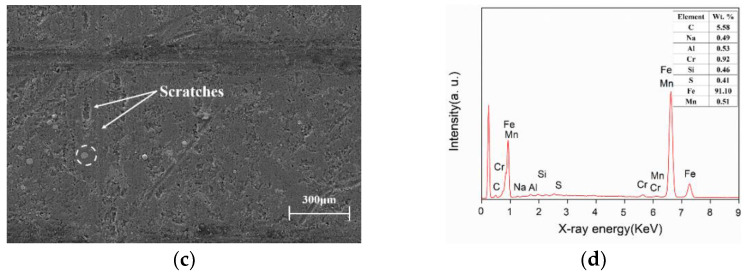
Typical SEM images and EDS spectra of the worn piston skirt under varied loads: (**a**,**c**) surface morphologies of the worn piston skirt under 8 and 10 kN, respectively; (**b**,**d**) EDS spectra of the wear debris observed in (**a**,**c**), respectively (circled in white).

Surface morphologies and EDS analyses of the severely worn areas of the piston skirts under 8 and 10 kN are shown in Figure 12a–d. The original valleys and plateaus, formed due to the interaction between the piston skirt surface and the surface of the cylinder liner, can be clearly distinguished from the micrographs, as shown in Figure 12a,c for 8 and 10 kN, respectively. The plane widths of the plateaus and valleys under 8 kN were about 270 and 30 μm, and about 280 and 20 μm under 10 kN. This indicated that the wear under 10 kN load was more serious than that under 8 kN. Round wear debris, from several micrometers to more than ten micrometers in diameter, adhering to the worn surface were also observed, as circled in white in Figure 12a,c. Formation of the round wear debris was believed to be the result of metallic fatigue in some reports [26,27], while it was considered to be due to adhesion at asperity contacts of tribo-pairs in some other studies [28,29,30]. EDS spectra of the wear debris are shown in Figure 12b,d. Significant levels of Fe that had not existed in the skirt material was detected, which indicated that adhesive wear occurred. Adhesive junctions are considered to form and break under high contact pressure, due to asperity contacts when a loaded skirt surface slides against the cylinder liner surface. In addition, the round debris were more evident under 10 kN, as shown in Figure 12c, compared to those under 8 kN, in Figure 12a. The results show that a higher load is more likely to cause adhesive wear, which is related to high contact pressure and local high temperature under higher loads.

**Figure 13:**
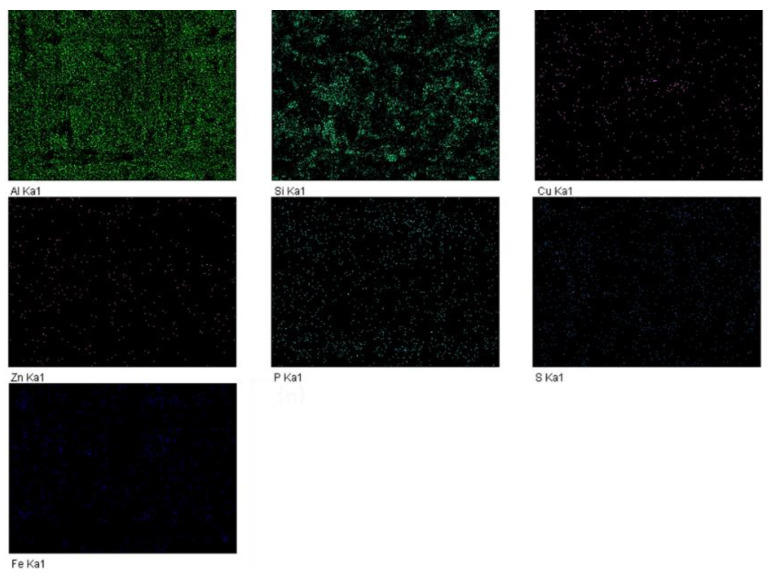
Element distribution of the worn surface.

Figure 13 shows the element distribution of the worn surface in Figure 12a according to EDS analyses. Al, Si and Cu are original elements of the piston skirt, while Fe is from the cylinder liner material due to wear. S and P distributed uniformly were most likely introduced by the ZDDP additives, and Zn may have come from both the original skirt material, as well as the ZDDP additives. In addition, Na that was found on the round wear debris, as shown in Figure 12d, was from the lubricating oil.

**Figure 14:**
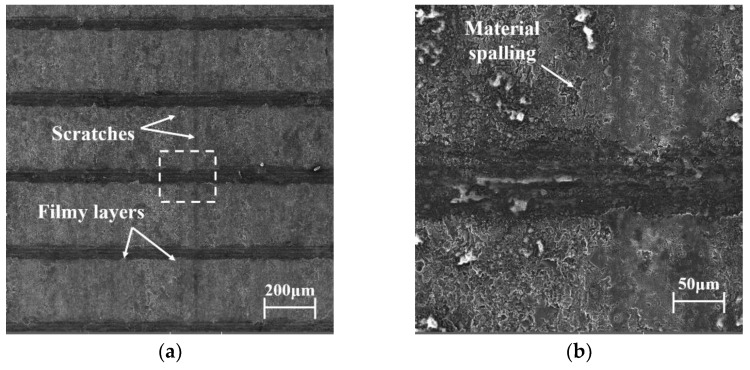
Surface morphologies of the piston skirt with relatively mild wear at different magnifications: (**a**) 200× magnification, (**b**) 1000× magnification.

Surface morphologies of the piston skirt with relatively mild wear are shown in Figure 14, which is helpful for studying the whole wear process. Figure 14b is a magnification of the rectangular area in Figure 14a. Traces of plastic deformation, as well as material spalling, due to fatigue wear, can be observed more clearly under high magnifications. Surface interactions in mixed or boundary lubrication conditions especially activate material plastic deformation in the contacting region. Scratches, considered a result of abrasive wear, were observed on the worn surface along the sliding direction, which were also found in Figure 11 and Figure 12. After a long time of reciprocating sliding, wear debris, generated due to fatigue and adhesive wear, were introduced in the interface and plowed grooves in the softer piston skirt surface. Abrasive wear would accelerate the wear of the piston skirt and the damage of the coating. It was noticed that plastic flow at the edge of the bearing plateau led to the formation of filmy layers, as shown in Figure 14a,b. The same phenomena were observed in former literature. The filmy layers were not obviously observed in the severely worn surfaces, as shown in Figure 11 and Figure 12, which is considered to be because the filmy layers separated from the plateaus after the serious wear process.

### 4.4. Wear Mechanisms of the Piston Skirt

**Figure 15:**
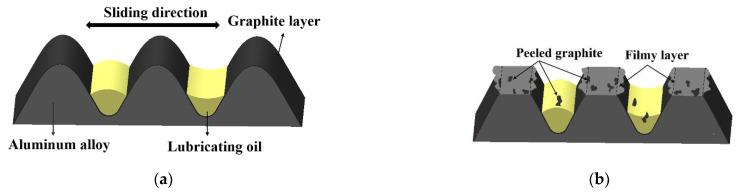

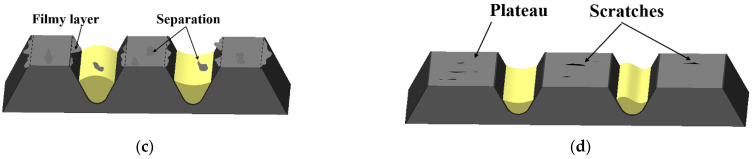
The simplified wear process of piston skirt sliding against cylinder liner: (**a**) running-in stage, (**b**–**d**) wear process.

Change of the piston skirt surface morphology during the wear process in this study is schematically shown in Figure 15. The unworn piston skirt surface had a typical profile after turning and was coated with a graphite layer. In the running-in stage, the load was light, only a small area of the graphite layer was in contact with the cylinder liner surface, and the lubricating oil was reserved in the valleys, as shown in Figure 15a. During the wear process, plateaus for bearing were generated due to the reciprocating sliding of the counter, as shown in Figure 15b–d. At the early stage of the wear process, the existence of the graphite coating aided in reducing the contact friction, thus, the friction force was low in the first 400 min, as shown in Figure 9. With the increase of the sliding cycles, graphite on the peaks totally peeled off and substrate materials began to make contact with the liner surface, as shown in Figure 15b. As the target load was high and the skirt surface was softer, formation of filmy layers occurred, due to plastic flow of materials on the edge of the plateaus. Filmy layers formed and then separated from the plateaus constantly as shown in Figure 15b,c. Finally, the height of the plateau was greatly reduced, due to wear, and the filmy layers at the edge of the plateau could be ignored, as shown in Figure 15d. The plateaus had a sufficient bearing capacity, and the wear rate became stable. On the other hand, the adhesion at asperity contacts of tribo-pairs induced adhesive wear. Some of the wear debris occurring during the whole wear process was involved in the friction surface and resulted in abrasive wear, which left grooves (scratches) on the worn surface, as shown in Figure 15d.

## 5. Conclusions

In this study, an apparatus that simulates the contact and the relative motion between the piston and cylinder liner in a real engine was designed and constructed. The effectiveness of the apparatus to investigate the friction and wear behavior of a cast aluminum alloy piston with a graphite coating sliding against a nitride cylinder was confirmed. The following conclusions were drawn:

1. The total wear of the piston skirt was higher under a higher load. The wear increased with increase of time in the initial stage, and then tended to be stable. In addition, the upper part of the skirt surface (around the height of the piston pin) was worn more severely, which is comparable to the results of a real engine.
2. The spalled graphite layer under contact stress acted as a solid lubrication film and aided in reducing contact friction at the early stage of wear. With the increase of the sliding cycles, solid lubrication films were removed or degraded, and an increase in friction coefficient occurred.
3. The main wear modes involved in the piston skirt wear process included abrasive, adhesive, and fatigue wear. Plateaus that were generated, due to plastic deformation, had a sufficient bearing capacity, which is thought to be responsible for stable wear after 8 h of the test. The round debris and scratches on the worn surfaces were evidence of adhesive wear and abrasive wear.
4. Whole piston and cylinder liner parts were tested within a comparatively short running time using the proposed apparatus that can truly reflect actual working conditions, which is helpful for a total understanding of the friction and wear behavior of the piston skirt.

## Figures and Tables

**Table 1:** The chemical composition of the piston.

Element	Si	Cu	Ni	Mg	Mn	Zn	Ti	Re	Al
Content (wt%)	11.5–12.5	3.0	2.5	0.5	0.5	0.2	0.2	0.35	Residual

**Table 2:** Experimental parameters.

Stage	Side Thrust Load (kN)	Temperature (°C)	Speed (rpm)	Lubricant Flow Rate (mL/min)	Time (h)
Running-in	0.2	120	200	2	1/3
Wear	8, 10, 12	120	200	2	40

## Data Availability

The data that support the findings of this study are available from the corresponding author upon reasonable request.
